# Defining the Recognition Elements of Lewis Y-Reactive Antibodies

**DOI:** 10.1371/journal.pone.0104208

**Published:** 2014-08-12

**Authors:** Somdutta Saha, Anastas Pashov, Eric R. Siegel, Ramachandran Murali, Thomas Kieber-Emmons

**Affiliations:** 1 Bioinformatics Graduate Program, University of Arkansas at Little Rock/University of Arkansas for Medical Sciences, Little Rock, Arkansas, United States of America; 2 Department of Biological Sciences, Research Division of Immunology, Cedars-Sinai Medical Center, Los Angeles, California, United States of America; 3 Stephan Angelov Institute of Microbiology, Bulgarian Academy of Sciences, Sofia, Bulgaria; 4 Winthrop P. Rockefeller Cancer Institute, University of Arkansas for Medical Sciences, Little Rock, Arkansas, United States of America; Cordelier Research Center, INSERMU872-Team16, France

## Abstract

Antibody response to carbohydrate antigens is often independent of T cells and the process of affinity/specificity improvement is considered strictly dependent on the germinal centers. Antibodies induced during a T cell-independent type 2 (TI-2) response are less variable and less functionally versatile than those induced with T cell help. The antigen specificity consequences of accumulation of somatic mutations in antibodies during TI-2 responses of Marginal Zone (MZ) B cells is a fact that still needs explanation. Germline genes that define carbohydrate-reactive antibodies are known to sculpt antibody-combining sites containing innate, key side-chain contacts that define the antigen recognition step. However, substitutions associated with MZ B cell derived antibodies might affect the mobility and polyspecificity of the antibody. To examine this hypothesis, we analyzed antibodies reactive with the neolactoseries antigen Lewis Y (LeY) to define the residue subset required for the reactive repertoire for the LeY antigen. Our molecular simulation studies of crystallographically determined and modeled antibody-LeY complexes suggests that the heavy-chain germline gene VH7183.a13.20 and the light-chain Vκ cr1 germline gene are sufficient to account for the recognition of the trisaccharide-H determinant Types 1–4, while the specificity for LeY is driven by the CDR3 backbone conformation of the heavy chain and not the side chain interactions. These results confirm that these monoclonals use germline-encoded amino acids to recognize simple carbohydrate determinants like trisaccharide-H but relies on somatic mutations in the periphery of the combining site to modify affinity for LeY through electrostatic interactions that leads to their optimized binding. These observations bring further attention to the role of mutations in T-cell independent antibodies to distinguish self from non-self carbohydrate antigens.

## Introduction

Antigen binding by an antibody is mediated by atomic interactions between the paratope (an antibody-combining site) and the epitope (antigen/determinant). An emerging concept, based on analysis of high-resolution crystal structures of carbohydrate-reactive antibodies and high throughput screening, is that germline antibodies often have polyspecific carbohydrate-binding paratopes and that Complementarity-Determining Regions (CDRs) may, contrarily to current paradigm, play only a secondary role in modifying the fine specificity of determinant recognition [1]–[5]. A corollary to this concept is that, for antibodies reactive with small carbohydrate forms, somatic diversification does not necessarily involve residues in direct contact with the antigen. The potential for somatic evolution of anti-carbohydrate antibodies has to be considered in the context of another phenomenon that has drawn attention - the restricted V region usage in many anti-carbohydrate responses - anti-Hib responses in humans [6], [7], anti-phosphatidyl choline responses in mice [8], anti-αGal responses in humans and mutant mice [9]–[11], among other examples.

The evolutionary conservation of preformed paratopes has been observed earlier for instance in studies on the anti-phosphoryl choline T15 idiotype [12], [13] and the nitrophenyl system [14] or more recently in the studies form on the germline anti-carbohydrate/hapten repertoire [15], [16]. The latter reports stress also the combination of a fixed specificity part of the paratope, which binds a relatively small structure and a flexible component, which allows for the accommodation of different flanking structures and further refinement of the specificity. In any case, these studies concentrate on a purely structural aspect and stay clear of discussing the immunological process that would ensure somatic affinity/specificity evolution and how this relates to the dynamical nature of the combining site that contributes to specificity. While some anti-carbohydrate responses can be thymus dependent, the majority is TI-2 and the current paradigm precludes their shaping by somatic hypermutation. B cells that express antibodies with highly polar or charged antigen binding sites and short CDR-3H facilitates entry into the marginal zone (MZ) compartment [17]. T cell-independent type 2 (TI-2) antigens induce rapid expansion of B cells in all areas of the spleen but the occasional Germinal Centers (GC) are abortive and the plasmablasts (PB) produced have low number of mutations like the predominant PB from extrafollicular origin [18]. T cell-independent responses typically depend on MZ B cells, which have the phenotype of memory cells in humans [19] and in mice they bare the signs of multiple rounds of reactivation [20]. All these reports find somatic mutations in TI-2 antibodies, which are low in numbers as compared to the antibodies gone through somatic hypermutation (SHM) but significant relative to the germline status.

To further the understanding of carbohydrate recognition by antibodies in these terms we reconsidered the restricted V gene usage and somatic adaptation, of antibodies to the neolactoseries carbohydrate antigen Lewis Y (LeY). The histo-blood-group Lewis antigens are cell-surface carbohydrates associated with a large number of human cancers, including lung, breast, colon and ovarian carcinomas. The Lewis antigens play an important role in tumor growth, progression and metastases [21] so they are of therapeutic interest [22], [23]. The LeY antigen is an embryonic antigen whose core consists of four hexose units that define the recognition element of LeY structures. The LeY core is highly restricted in its conformational properties associated with the H-type 2 determinant, L-Fuc–α1, 2Gal-β1,4GlcNAc [24]. Along with several modeled anti-LeY recognition schemes being presented for the monoclonal antibodies B3, and BR55-2 [24], [25], crystal structures of LeY-reactive antibodies are available that include the murine antibody BR96 [26], and the humanized antibody 3S193 [27].

The LeY antigen is closely related to the murine embryonic antigen SSEA-1 (LeX) in its carbohydrate structure. LeX is a self-antigen in mice [28]. Here, we demonstrate that the germline V-gene family VH7183.a13.20, along with the Vκ *cr1* germline gene, encodes the key structural features defining the recognition of the footprint of the trisaccharide-H determinant Types 1–4. Most notably, the LeY-reactive antibodies present an example in which the somatic mutation does not involve residues in direct contact with the LeY antigen but the substitutions lend to both a restriction and increase in antibody flexibility relative to the germline precursor. This suggests that these antibodies are most likely derived from low intensity mutation and selection process outside germinal centers that yields with high probability the desired specificity distinguishing between self and non-self carbohydrate epitopes by modulating structural flexibility and relying on electrostatic interactions with the LeY antigen.

## Methods

### Antibody modeling

The structure of the binding site was addressed using computer-simulated molecular models generated by homology modeling [24], [25], [29]. Computer-assisted homology-based modeling provides a reliable method of defining the three-dimensional structure of the binding pocket. Using this methodology, antibodies whose structures are defined by crystallography can be used as a template for structural studies on an antibody of interest. The sequence alignment of the Variable (V) region of the Light (L) and H (Heavy) chains was performed using CLUSTALW *(* www.ebi.ac.uk/clustalw). The Variable regions of the four antibodies were structurally aligned using the Align Structures Module of the Protein Modeling Protocol in Discovery Studio (DS) 3.1 (Accelrys Inc., San Diego, CA). The murine form of hu3S193 was constructed starting from the humanized structure, using the ‘Build Mutant’ protocol in DS to perform the step-by-step mutation of the humanized form in reverse order while calculating the Root Mean Square Deviation (RMSD) of the new structure at each step against the preceding structure. This was done to ensure that the conformation of the antibody does not change drastically compared to a single-step all-mutation process.

The germline antibody model was built by identifying a suitable template structure based on sequence homology. The putative germline sequences were obtained by IgBLAST (http://www.ncbi.nlm.nih.gov/igblast/) search with V region of the L and H chains of each murine protein separately as the query sequence. The V_H_ and V_L_ sequences ranked highest by E-value were chosen as the candidate sequence for the germline V_H_- and V_L_-model building. Single amino acid substitutions were made on the available mature murine structures (either the crystal structure or modeled structures) using DS’s ‘Build Mutant’ protocol, which resulted in the final germline antibody structures. At each mutation step, an all-atom-based RMSD calculation was performed against the initial structure to check conformational stability. RMSDs were calculated and compared among the germline structures differing in their starting structure to look for robustness of the modeling software [29].

### Molecular Simulations

Calculations were performed under *in vacuo* conditions on each of the five antibodies in both their bound and free conformations. The starting models for the simulation were from crystal structure studies and, in some cases, structures were homology-modeled in the lab. Solvents from crystallographic models, ions and heteroatoms were removed except for the bound LeY. Hydrogen-atom positions were assigned using DS. Prior to Molecular Dynamics (MD) simulation *in vacuo*, the structures were energy-minimized with 2000 cycles of Steepest Descent followed by another 1000 cycles of minimization using the Conjugate Gradient method to eliminate close contacts between atoms [24], [25], [30]. The energy minimizations were followed by 300 picoseconds (ps) of MD simulation where the system was slowly heated to 300K and then equilibrated at a constant temperature of 300 K for 250 ps. The MD simulations were carried out using the CHARMM force field with a time step of 1 fs without using any restraints. This was then followed by a production run of 300 ps for each system. A 14A° non-bonded list radius and a spherical cutoff for electrostatics (the solvent dielectric constant being 1) were used in both the bound and free conformations of the antibodies.

The resulting individual structures of the ensemble were evaluated for their total energy and how close each was to the initial starting structure based on an all-atom RMSD calculation. The lowest-energy structure with the lowest RMSD was picked for further analysis. The RMSD fluctuations were calculated using the ‘Analyze trajectory’ protocol in DS. The Van der Waals contacts for the final structures were determined using a cutoff of 4 A°. The potential intermolecular hydrogen bonds were listed out by ‘Intermolecular Hydrogen Bond’ monitor in DS and also visually confirmed. The interaction energy between the antibodies and the LeY was calculated using the ‘Interaction Energy’ protocol in DS. In some cases, individual interaction energies with respect to the amino-acid residues were also calculated. Individual H- and L-chain contributions towards antigenic interaction were also determined for some specific cases.

### Restrained dynamics

The monoclonal antibodies were also subjected to restrained dynamics where residue-specific harmonic and distance restraints were used. MD calculations were performed first by restraining those germline residues using a distance restraint that were observed in the human anti-LeY antibody crystal structure to participate in intermolecular hydrogen bond interactions with LeY (pdb code: 1S3K). This was repeated next by restraining those residues that the germline model of the human anti-LeY antibody picked up for intermolecular hydrogen bonding with LeY at the binding pocket. All calculations were conducted *in vacuo* by setting the dielectric equal to one. While minimization with charged residues in the absence of a solvent (physiological conditions) is very unrealistic and is sure to move a structure far from its X-ray-determined position, we compensated for this effect by restraining interaction sites corresponding to an X-ray-determined position [27]. The inter-molecular hydrogen bonds and the corresponding non-hydrogen atomic distances between the participating acceptors and donors were calculated for the antibodies using the final conformation obtained after standard dynamics. Intermolecular hydrogen bonds were listed out, and interaction energies were calculated for further comparison.

### Statistical Analysis

In each murine antibody considered, the nucleotide changes from the germline sequences were individually classified (1) by region as FR (framework region) versus CDR (see above) according to their locations in the V_H_ or V_L_ domain, and (2) by mutation type as R (replacement) versus S (silent) according to their effects on the antibody’s amino-acid residues. The resulting data for all four antibodies were then analyzed for whether R mutations occurred significantly more often in one region compared to the other, using a stratified-analysis approach with each antibody as a stratum, in order to prevent bias due to Simpson’s Paradox [31]–[33] Specifically, we used Cochran-Mantel-Haenszel (CMH) methods [31], [32], namely, the Mantel-Haenszel Common Odds Ratio (OR) to estimate the magnitude of association averaged across all strata (i.e., across the four antibodies), the CMH chi-square test of association to test the significance of the common OR, and the Breslow-Day chi-square test of association homogeneity to test the validity of estimating a common OR across strata. Separate analyses were conducted for the V_H_ and V_L_ domains.

## Results

### Sequence Alignment

**Figure 1:**
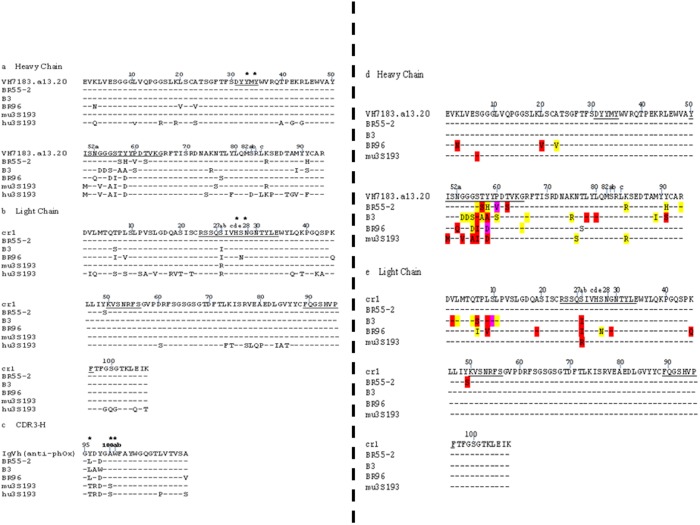
Sequence alignment of the variable domains of the (a) Heavy chains, (b) Light chains, and (c) homology among the CDR3-H of anti-phOx germline antibody, BR55-2, BR96, B3, mu3S193 and hu3S193. Dashes indicate identities with respect to the germline antibody sequence. Numbering corresponds to that of Kabat, CDRs are underlined. Asterisks indicate amino acid residues that participate in antigen contact based upon hu3S193 crystal structure. (d) Heavy chain and (e) Light chain amino acid sequence alignment showing the distribution of nucleotide substitution(s) -the amino acids have been color coded representative of the nucleotide substitution(s) found at that position with respect to the germline sequence– yellow: transition type substitution at any of the nucleotide positions comprising the codon, red: transversion type substitution at any of the nucleotide positions comprising the codon, pink: when both transition and transversion substitutions occur in the codon for that corresponding amino acid. Footnote: Position 77H in BR96 is Serine in its PDB entry but it corresponds to Threonine in the nucleotide sequence as reported in its US patent no: 57288211. Hence, the type of nucleotide substitution could not be determined at that position. Also, gene sequence for cr1 could not be found beyond position 94. Nucleotide substitutions beyond that were not considered.

The murine V_L_ and the V_H_ domains (Figure 1) of the four murine antibodies considered here – BR55-2 (IgG3), B3 (IgG3), BR96 (IgG3), and mu3S193 (IgG3) are highly homologous with the VH7183.a13.20/VH50.1 (Figure 1a) and Vκ cr1 germline (Figure 1b) gene families. The CDR3 region of the heavy chain, on the other hand, is highly homologous to the IgVH anti-phenyloxazolone gene (Figure 1c) and also has the second highest number of non-synonymous mutations. When compared against the germline nucleotide sequence, the majority of the observed substitutions in the murine antibodies occurred in the heavy-chain CDR2 region (Figure 1a,d), followed by the flanking framework regions, FWR1 and FWR3. In this analysis, all instances of base substitution(s) in a codon have been considered for comparison purpose. Predominantly, transversions occur more than transitions in both chains. Table 1 shows the results of classifying mutations in each antibody’s V_H_ domain by region and mutation type. Analysis via CMH methods of the Observed R/S ratios in Table 1 revealed a statistically significant association of mutation type with V_H_ region. In the heavy chain (Figure 1d), BR96 has the highest number of transversions, seven, with five concomitant amino-acid substitutions, whereas mu3S193 and B3 have six transversions each, with five and two associated amino-acid substitutions, respectively. BR55-2 has the least number of transversions among the antibodies (three) with concomitant amino-acid substitutions. On average, the odds of having an R mutation were more than six-fold higher in the CDRs than in the FRs (common OR = 6.26, CMH chi-square = 3.92, DF = 1; *P* = 0.048). Moreover, this result was accompanied by a non-significant Breslow-Day test result (chi-square = 1.08, DF = 3; *P* = 0.78), which indicates that the individual OR of each antibody does not differ significantly from the common OR of 6.26.

**Table 1:** The occurrence of mutations in the hypervariable region of the V_H_ chain of the monoclonal antibodies when compared against their germline gene sequence and the observed R (Replacement) and S (Silent) mutations.

Mab V_H_ Chain	Mutations,FR1		Mutations, CDR1		Mutations,FR2		Mutations CDR2		Mutations FR3		Total Mutations- FR		Total Mutations- CDR	
	R	S	R	S	R	S	R	S	R	S	R	S	R	S
BR55-2	0	0	0	0	0	0	4	1	2	1	2	1	4	1
B3	0	0	0	0	0	0	6	1	3	3	3	3	6	1
BR96	3	0	0	0	0	0	4	0	0	1	3	1	4	0
mu3s193	0	1	0	0	0	0	5	0	2	0	2	1	5	0

Regions beyond FR3 are not considered for the analysis as they form the junction region and the origin of the junction regions cannot be predicted accurately [57] (FR = Framework Region, CDR = Complementarity Determining Regions).

For light chains (Figure 1e), more transversions are also observed in the gene sequences. B3 has the highest number of transversions (six) followed closely by BR96 (five), each having two associated amino-acid substitutions. BR55-2 and mu3s193 each have one, with one concomitant amino-acid substitution per clone. A similar analysis via CMH methods of the observed R/S ratios in each antibody’s V_L_ domains (data not shown) was also supportive of increased odds favoring R mutations in CDRs (common OR of 2.42), but the increase was not significant for V_L_ (chi-square = 0.62, DF = 1; *P* = 0.43). Overall these results suggest that transversion type mutations, associated with affinity maturation, are observed in these antibodies despite their evident TI origin.

### *In vacuo* recognition of LeY by the germline antibody

The sequence comparison of the germline sequence with the crystallographically determined anti-LeY antibodies provides a template for the variable region of the germline sequence. Super-positioning of the heavy and light chains of the respective antibodies suggest good structural similarity between the four antibodies, and the relative orientation of the V_H_ and V_L_ domains is the same in the bound conformation for all four antibodies being compared. RMSD values observed to be within 0.5Å were calculated taking into account the backbone Cα atoms and a pairwise comparison of the bound conformations were also made. The CDRs were also separately structurally aligned following a pairwise superimposition. The LeY antigens from the four antibodies were also individually superimposed to further ensure that they are all structurally similar.

The germline model antibody was constructed in accordance with the homology-based modeling approach and the resultant model structure of the germline sequence was subjected to energy minimization and MD simulations. To determine the relative importance of individual amino acid replacements that might affect antibody structure, a BLAST search of the VH7183.a13.20 gene on structurally known antibodies was performed. High levels of homology were observed with the several crystallographically resolved antibodies. The highest homology level (99%; 97/98 residues) was with an anti-canine lymphoma monoclonal antibody (Mab231) [34]. The one mutational difference occurs in Framework 1. The CDR3-H of the VH7183.a13.20 gene is a 15-residue long loop and displays a K+ take-off geometry based on main-chain conformation of the loop and the quadrant it occupies in the Ramachandran φ,ψ map [35]. The K+ type of arrangement has a bulge-like conformation with the Ramachandran code (bab) [36] at residues G-7 (Gly 99), G-6 (Ala 100) and G-5 (Trp 100a) in addition to the kinked plus bulge (K+), which is shared with the heavy chain of the anti-phenyl-oxazolone antibody as a starting template for the monoclonals.

**Figure 2:**
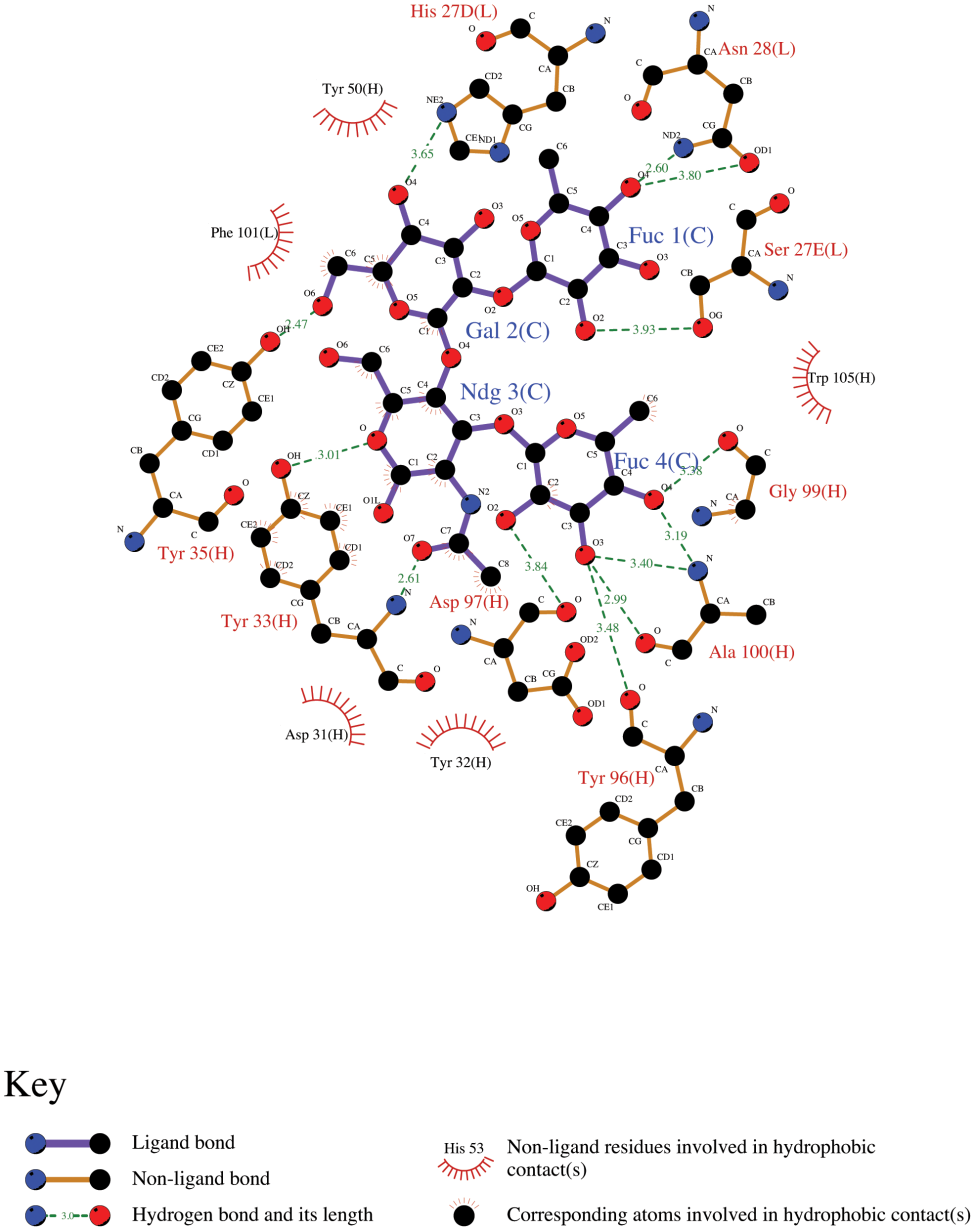
LIGPLOT [59] of LeY recognition by the germline model structure where Fuc represents α-l-fucose, Gal: β-d-galactose and Ndg: N- acetyl D-glucosamine.

Based upon the LeY-crystal structures and sequence homologies, the germline polar contacts involve hydrogen-bonding residues that include the side-chain interactions of L-His 27D and H-Tyr 35 contacting Gal, side-chain interactions of L-Asn 28 and L-Ser 27E contacting Fuc1, the side chain of H-Tyr33 and its backbone atoms contacting NAG, the backbone interactions of H-Tyr 96, H-Asp 97, H-Gly 99 and H-Ala 100, contacting Fuc4 (Figure 2). The interactions of H-Tyr 35 and L-His 27D with Gal are conserved in all four of the murine antibodies and in the humanized form. In this context, a central feature of the germline paratope seems to be a pocket for the Gal moiety to be built upon as a main prerequisite for bridging the germline heavy and light chains. Built around this pocket the side-chain hydrogen bonds involving L-Asn28 and H-Tyr33 define the recognition of the trisaccharide core Fuc(1,2)-Galβ1-2GlcNAc, with this recognition scheme for the glycan structure conserved in all of the antibodies, whereas the Fuc4 structure is stabilized by a series of backbone interactions which are predicated on a canonical conformation of CDR3-H.

Another site of substantial change is the CDR3-H region, where some of the germline antibody’s contact residues have undergone somatic mutation. This is in keeping with arguments that the VH CDR3 loop exerts the greatest influence on antigen-binding specificity [37]. The CDR3 H-domain sequences of BR55-2 and BR96 are close to the germline sequence, with only one and two residue substitutions, respectively (Fig. 1c). Based upon the crystal structure of BR96, H-Ala100 contacts Fuc4 through backbone interactions. H-Ala100 is conserved in all antibodies except for the 3S193 antibody, which has a Ser substitution at this position.

### *Energetic basis* of LeY recognition

To determine if mutational bias influences amino acid patterns that affect recognition and affinity for LeY, a comparative-dynamics study was performed, followed by component analysis to provide detailed mechanistic insight into the antigenic recognition and binding interactions for the LeY antigen by the germline sequence relative to the other anti-LeY antibodies. To compare the efficiency of the current method of estimating free energies of binding to an experimental approach, we compared our calculated interaction energies between LeY and the respective antibodies with published values of the dissociation constant (K_D_) for the interaction (Table 2); K_D_ values for mu3S193 and hu3S193 were taken from [38], BR96 from [39]and fro BR55-2 from [40].

**Table 2:** Calculated total interaction energy and its two components – total Van der Waal’s Energy and total Electrostatic Energy.

Antibody-Carbohydrate Complex	Total Interaction Energy (in Kcal/mol)	Total Van der Waals Energy (in Kcal/mol)	Total Electrostatic Energy (in Kcal/mol)	K_D_ (in M)	Total No of mutations in framework region with respect to the germline structure	CDR2-H electrostatic contribution (in Kcal/mol)
mu3S193-LeY	−226.87	−15.18	−211.69	5.3×10−9	9	−80.55
hu3S193-LeY	−220.15	−15.61	−204.54	2.4 ×10−7	51	−67.15
BR96-LeY	−213.13	−11.56	−201.56	9.9 ×10−6	13	−87.39
Germline antibody	−212.84	−15.25	−197.59	N/A	0	−83.21
B3-LeY	−211.64	−13.37	−198.27	N/A	11	−95.30
BR55-LeY	−188.16	−10.54	−177.62	8×10−6	6	−110.21

The K_D_ values are taken from literature for comparison purpose.

The mu3S193 with 9 and BR55-2 with 7 non-synonymous mutations in their framework region form the topmost and bottom-most member, respectively, in the avidity scale (K_D_) (Table 2). As noted, our calculations trend with published, experimentally observed K_D_s.

Component analysis of the calculated free energies of binding suggests that the Van der Waals interactions are close to being optimal before somatic mutations. In contrast, electrostatic interactions between LeY and the antibodies was the dominant factor for binding affinity and K_D_ trend. The most predominant change in amino acid residues occur at L-Ser 27E, which is conserved in all antibodies except for BR96. The emphasis on electrostatics in the interactions would suggest that the transition-type substitution of Asn for Ser at position L-27E, as found in BR96, would increase the binding energy for LeY. Indeed, this substitution in the germline antibody model results in an interaction energy of −232 Kcal/mol and no significant change in the Van der Waals energy component (−16.89 Kcal/mol) (Table 3).

**Table 3:** Calculated total interaction energy for germline structures with single non-bond residue contact substitutions to Alanine and Arginine for position 27E.

Antibody-Carbohydrate Complex	Total Interaction Energy (in Kcal/mol)	Total Van der Waals Energy (in Kcal/mol)	Total Electrostatic Energy (in Kcal/mol)
Germline-ab(L-Ser27E ->Asn)	−232.21	−16.89	−215.32
Germline-ab (H-Trp100A −>Ala)	−218.02	−15.460	−202.55
Germline-ab (H-Asp31 −>Ala)	−212.95	−15.23	−197.72
Germline antibody	−212.84	−15.25	−197.59
Germline-ab (H-TYR50−>Ala)	−200.23	−15.23	−184.99
Germline-ab (L-Phe101−>Ala)	−201.30	−15.16	−186.14
Germline-ab (H:Tyr32 −>Ala)	−192.63	−15.23	−177.40

**Figure 3:**
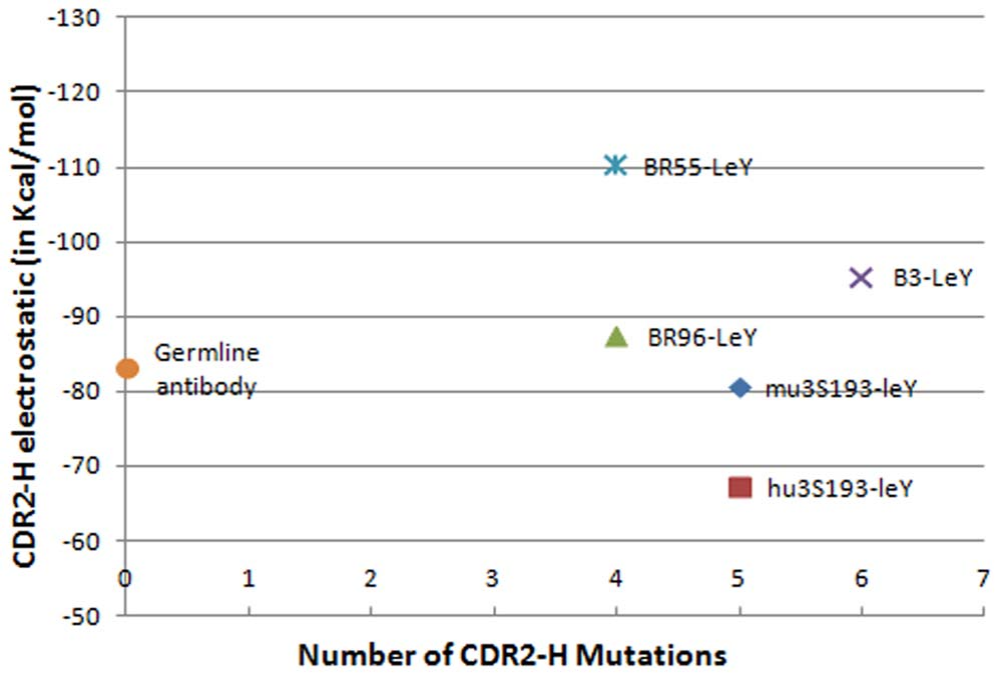
The occurrence of CDR2-H mutations in the antibodies and its electrostatic contribution in binding LeY.

Since the majority of the mutations were found to occur in the CDR2-H region, and since the Van der Waals component of the total interaction energy is already optimized from the germline to the mature antibodies, it becomes important to analyze the electrostatic contribution to the total interaction energy of the antibody-antigen complex. The interaction-energy calculations between our antibody-LeY complexes and their respective CDR2-H regions (Table 2, Figure 3) show that BR55-2 has the highest electrostatic CDR-2 contribution, and BR96 the 3^rd^ highest, even though they are tied for having the fewest number of mutations (four) in this hypervariable region, whereas B3 has the 2^nd^ highest CDR-2 contribution even though it has the most number of mutations (six) in this region. These results suggest that there is no direct correlation between the number of mutations and the electrostatics –an argument in favor of the existence of multiple short pathways for the somatic evolution of these antibodies.

### Antibody Flexibility versus Rigidity

**Figure 4:**
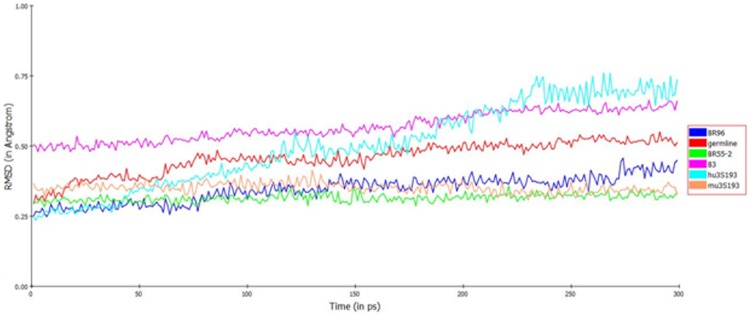
RMS fluctuations of the germline and mature antibody-LeY complexes. The RMS fluctuations are calculated after superimposing the backbone atoms of each conformer (as generated from the MD production run) against the average structure.

To determine if the few somatic mutation optimized the binding-site conformation for LeY recognition, we compared the degree of conformational diversity between the various states of the antibodies generated during the MD simulation. Analyzing the backbone RMSD from the average structure over the course of the trajectory illustrates an antibody’s stability and plasticity. The RMS fluctuations for the mature antibody-LeY complexes were compared individually with respect to the RMS fluctuation curve of the germline antibody-LeY complex over time. As shown in Figure 4, atoms in the germline complex displayed greater fluctuations (RMS 0.29 Å to 0.52 Å) than those in the mature complex (0.25 to 0.39 Å) of three of the antibodies. These results suggest that the germline complex has greater flexibility than BR96, BR55-2 and mu3S193, in keeping with the current paradigm that germline antibodies are more flexible. In contrast, B3 and the humanized version of 3S193 displayed greater fluctuations than that of the germline antibody. Significant RMSD fluctuations observed for B3 and hu3S193 in the initial few hundred ps of the MD run suggest that the structures reorient themselves at the paratope interface, while still retaining an overall structural stability. These results suggest that motions at the domain interface might dynamically impact the conformation of the paratope-binding surface of these antibodies, and may provide an adaptive separation distance necessary to interact with LeY.

## Discussion

LeY-reactive antibodies are associated with the VH7183 family, suggesting that they arise from the mouse natural autoantibody repertoire [41]. Germline antibodies are polyspecific due to the structural flexibility of their preformed binding site. The sequence relationship between mu3S193 and BR55-2 relative to their antigen affinity demonstrates the fact that only a few mutations are needed to convert family members into potent antigen binders. Our calculations suggest that the germline sequence displays optimal (hydrophobic) interactions for recognition of LeY but suboptimal electrostatic interactions. In other words, a small hydrophobic anchoring of core residues are nucleated by the germline antibody, followed by dynamic polar interactions that enhance the affinity and specificity of the binding process. Similar results have been found in the analysis of the Kdo recognition by anti-LPS antibodies [16]. The observation of a germline-coded pocket for the recognition of the Gal core of LeY suggests a pathway for specificity; the presence of a small recognition site within a flexible variable domain represents a strategy whereby the germline antibody pairing of heavy and light chain could specifically bind to the core and then, following mutations, yield diverse LeY-reactive antibodies. However, the LeY-reactive mature antibodies use most of the germline-encoded amino acid residues for binding of LeY.

Greater affinity is accomplished through the optimization of electrostatic interactions in the binding site to fine-tune the nuances of the antigen’s fine structure (e.g. stereo-specificity in LeY). The observed R/S ratios for CDRs in the monoclonals indicate that these regions are more prone to R-type mutations than the framework regions despite their evolving from T-cell independent events as typically thought of for T cell-dependent (TD) antigens. When compared against the inherent R/S ratio [42] for a random sequence (which is 2.9, the number most often used to denote somatic hypermutation), it was found that the FRs have higher R/S. The CDRs displayed a greater-than-expected frequency of R mutation [43], [44] than did the FRs. Thus, the high R/S ratios may be inherent to the CDR sequences rather than a sign of evolutionary pressure and this analysis points to the fact that the changes do not necessarily reflect a somatic hypermutation based maturation process [44].

We also observe that small structural changes in a binding site can completely change the balance between the enthalpy contributions of the scaffold and directional interactions that enable a variety of different structural solutions to be explored to achieve the same functional outcome. Changes in the electrostatic contribution in interaction with a ligand need not involve contact or recognition site residues, nor is one substitution type more important than another.

For antibodies against T-cell-dependent antigens, the acquisition of high affinity to antigens is known to proceed via antigen-driven evolution based on somatic hypermutation [45], [46]. Anti-carbohydrate responses, though, are typically thymus independent and the product of highly compartmentalized B cell populations – MZ B cells and B1 cells to some extent. Thymus independent responses are fast, mostly of IgM and IgG3 isotypes, lack somatic hypermutation process and memory establishment. Their dynamics, though, has earned them the label “natural memory” [47]. The TD and TI mechanisms may be differentiated through indirect signs like the isotype of the antibodies, the degree of the somatic mutation and the evidence of antigen driven adaptation. On the other hand, affinity maturation of carbohydrate antibodies is not excluded, nor is thymus-dependent responses under certain circumstances [48]. MZ B cells are competent both for class switch recombination (CSR) and SHM [49]. As a rule they carry mutated V regions and, especially in humans, the diversification seems to happen very early in the ontogeny [50].

In some species somatic diversification exists also outside of the germinal center microevolution and instead of adapting the BCR to the selecting antigen it rather ensures globally the completeness of the repertoire prior to the antigen encounter [51]. Apart from an atypical thymus dependent response, the presence of somatic mutations in anti-carbohydrate antibodies could be the sign of a similar process of variable region diversification of T cell-independent marginal zone B cells [50], [52]. Although marginal zones do not seem to exhibit AID activity except in a minor population of B cells [53], the MZ B cells may have a very different dynamics of diversification driven by a low level of AID expression only during cycling [52]. Recently a role for B-helper neutrophils was reported in inducing AID in marginal zone B cells [54]. Weller et al. have proposed also a model of development of the human counterpart of the MZ B cells involving a slow buildup of relevant clones stimulated through TLR10 and accumulating somatic mutations independent of antigen stimulation [50], [55]. Furthermore, when HEL specific follicular (FO) and MZ B cells were separately adoptively transferred, MZ proved slower to enter follicles and to mount SHM but had a normal rate of CSR [49]. Thus, the differentiation of marginal zone B cells is not due to the inability for mounting TD responses. Rather, they are forced in a program ensuring specific dynamics of their somatic mutations and antibody production related to their repertoire [17], [56]. Since, like B1 cells, MZ B cells are partially self replenishing “natural” memory cells that undergo multiple cycles of reactivation and division, a slow “tick over” diversification in step with their reactivation cycles could underlie a stochastic process that sometimes amounts to short evolutionary path adaptation through low frequency mutation.

The hypothesis of an alternative way for adaptation of TI-2 antibodies is complementary rather than antidogmatic. The current paradigm considers only two alternatives: TD responses with SHM and TI-2 responses without a SHM adaptation. Anything in between is considered a misclassified case of one of the above. Repertoire generation and affinity maturation are different outcomes of somatic mutation process. Yet, in the LeY antibodies studied there are signs of adaptation of the paratope to the epitope through somatic mutations. Apart from the possibility of TD GC process, unlikely in view of the IgG3 isotype of the studied antibodies and the low level of mutations, is it possible that the “tick over” mutation process leads to an affinity selection happening over several cycles of reactivation of the respective MZ B cell clones? This process may be orders of magnitude slower than the GC reaction but in line with the dynamics of the formation of the MZ compartment.

LeY and LeX are very similar. Antibodies raised against LeY expressing cells show a typical TI-2 profile and rarely cross-react with LeX while those raised against synthetic LeY coupled glycoproteins are TD and cross-react with LeX [57]. Thus, the natural TI-2 response to LeY differentiates between LeY and the very similar self-antigen LeX. May be the hard problem of tolerance maintenance in the grey zone of high cross-reactivity with self-carbohydrates has been solved by combining preformed paratopes encoded in the germline repertoire, followed by a meticulous and slow selection of the right clones to enter the MZ compartment. It would be interesting to check the hypothesis that an equally slow and cautious paratope adaptation occurs over a time period comparable with the length of the individual live.

The VH7183 germline antibody family, to which the LeY-reactive antibodies belong, is likely to possess a polyspecific-binding site. It is possible that evolution of the germline immunoglobulin genes favored preformed flexible paratopes like that of VH7183, which are only a couple of mutations away from several different biologically relevant specificities. Similar maturation can be easily and consistently achieved during the slow (non-SHM) somatic diversification due to the presence of low level of AID in the early stages of the development of the TI B cell population [50]. Here it was been shown that, at least in the case of murine LeY antibodies, this strategy is related to mutations away from the binding site – reminiscent of non-specific epistasis [58]. These studies also suggest that antibodies with increased flexibility introduced either by humanizing or thorough evolution might lead to unwarranted side effects if one considers the paradigm that germline antibodies are polyspecific because of their inherent flexible nature.

## Conclusion

Our studies show that germline LeY reactive B cells yield higher affinity clones as a result of a somatic diversification. The germline antibody is preformed to recognize salient glycan fragments of LeY. Accumulating discrete mutations as part of a process of diversification that has only recently been addressed yields with high probability clones that accommodate better the fine structure of the antigen fragments. This reflects a strategy different from germinal center related somatic hypermutation for exploration of the paratope space close to the limit of tolerance, as target and self-carbohydrate antigens are naturally highly cross-reactive. While this study highlighted the occurrence of anti-LeY thymus independent antibody related to a single germline V gene family, it might be true to some extent for other types of TI- 2 antigens as well. Thus, as our knowledge about the structural details of antibody-antigen interaction broadens, we may be able to design better antibodies, as well as design improved antibody based vaccines through more refined reverse-engineering concepts based on the knowledge of antibody structural plasticity and specificity, paving the path towards improved immunotherapy for cancer patients.
